# Supplementary material for: Beneficial effects of climate warming on boreal tree growth may be transitory
Source: Nat Commun. 2018 Aug 10;9:3213. doi: 10.1038/s41467-018-05705-4 (PMC6086880; doi:10.1038/s41467-018-05705-4)
Supplement: Supplementary file 1 — Supplementary Information [file 41467_2018_5705_MOESM1_ESM.pdf]

Supplementary Information

Beneficial effects of climate warming on boreal tree growth may be transitory (D'Orangeville et al.)

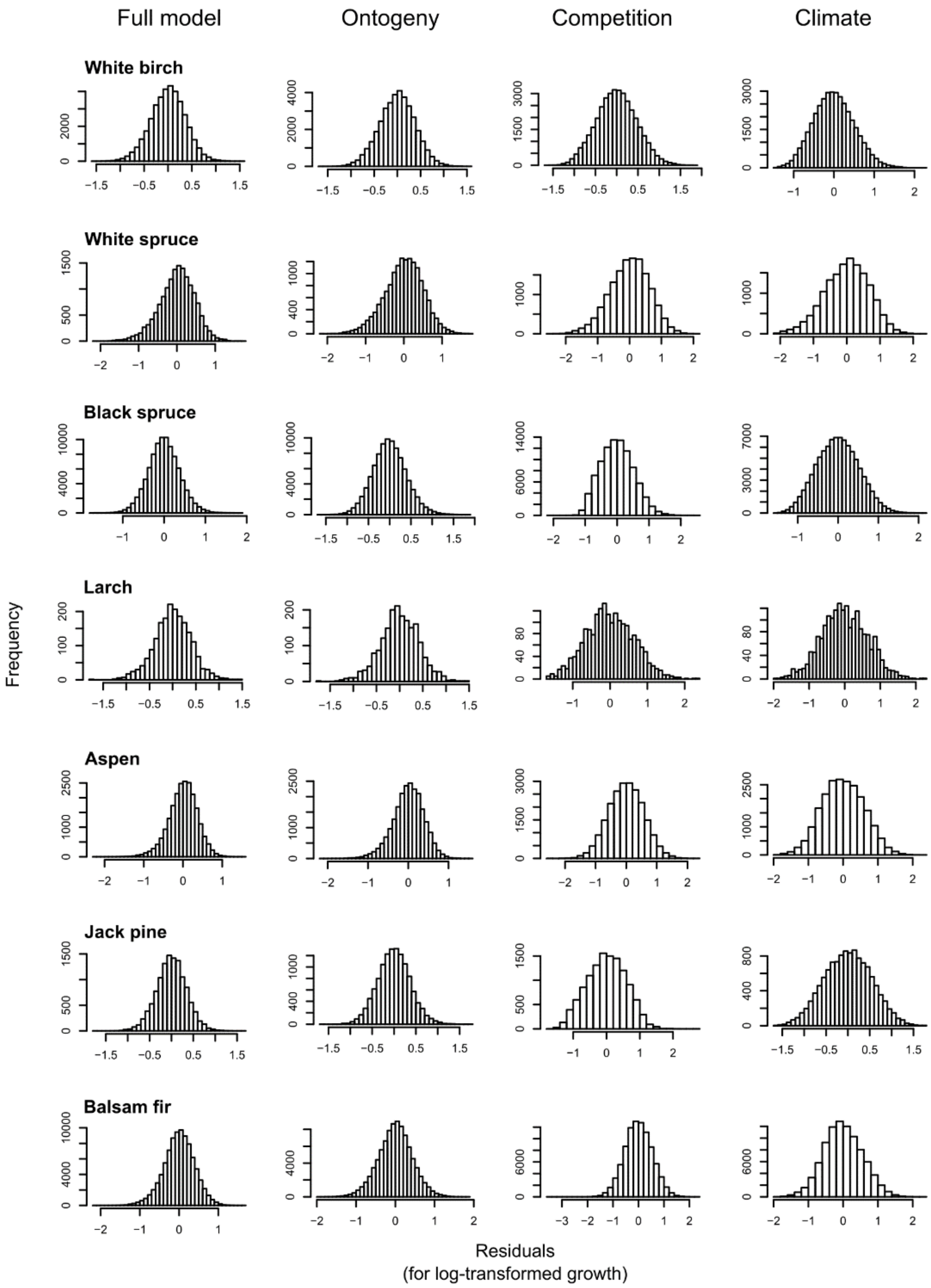

Supplementary Figure 1 Distribution of full and partial model residuals. Partial models consist of i) ontogeny effects (tree size and age), ii) competition effects (symmetric and asymmetric competition) and iii) climate (temperature, water availability and snowpack).

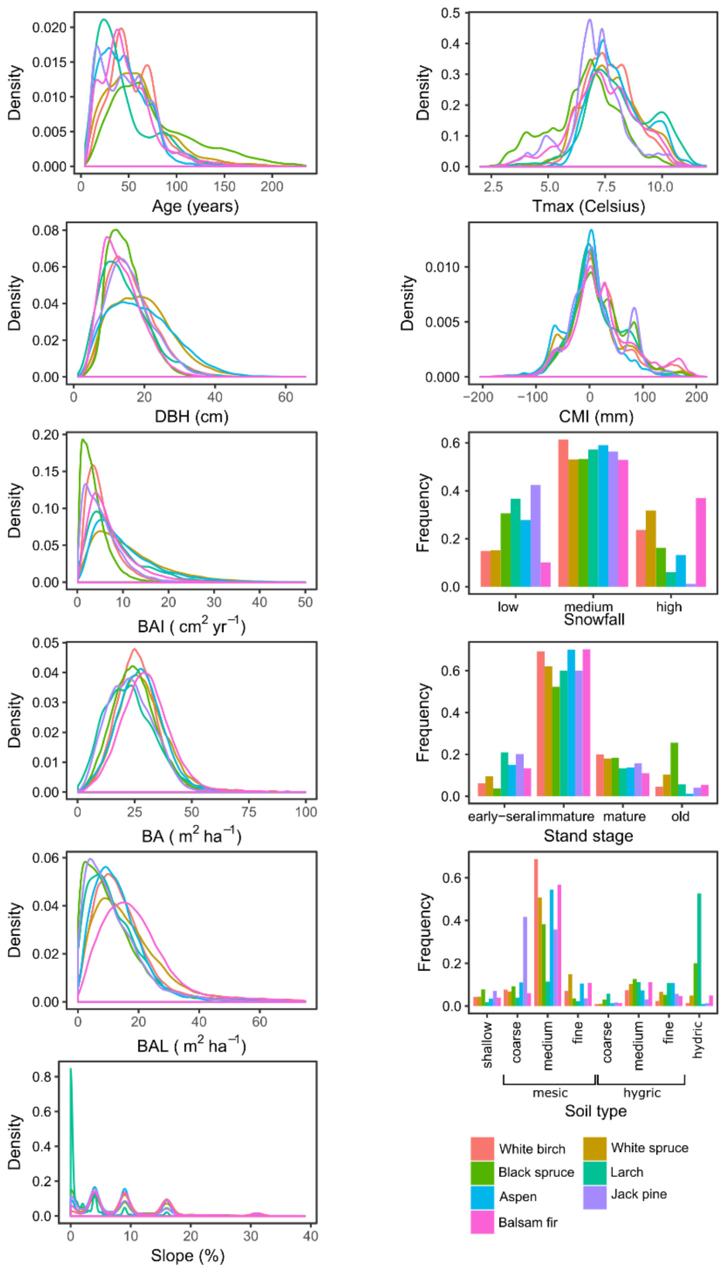

Supplementary Figure 2 Distribution of the variables included in each species growth models using Kernel density estimates. Note that the shape of the density estimate for terrain slope is due to the combination of sites with continuous slope measurements and sites with slope measurements aggregated into semi-quantitative classes (set to their lower range value, i.e. 0, 4, 9, 16, 31 and 41%; see methods).

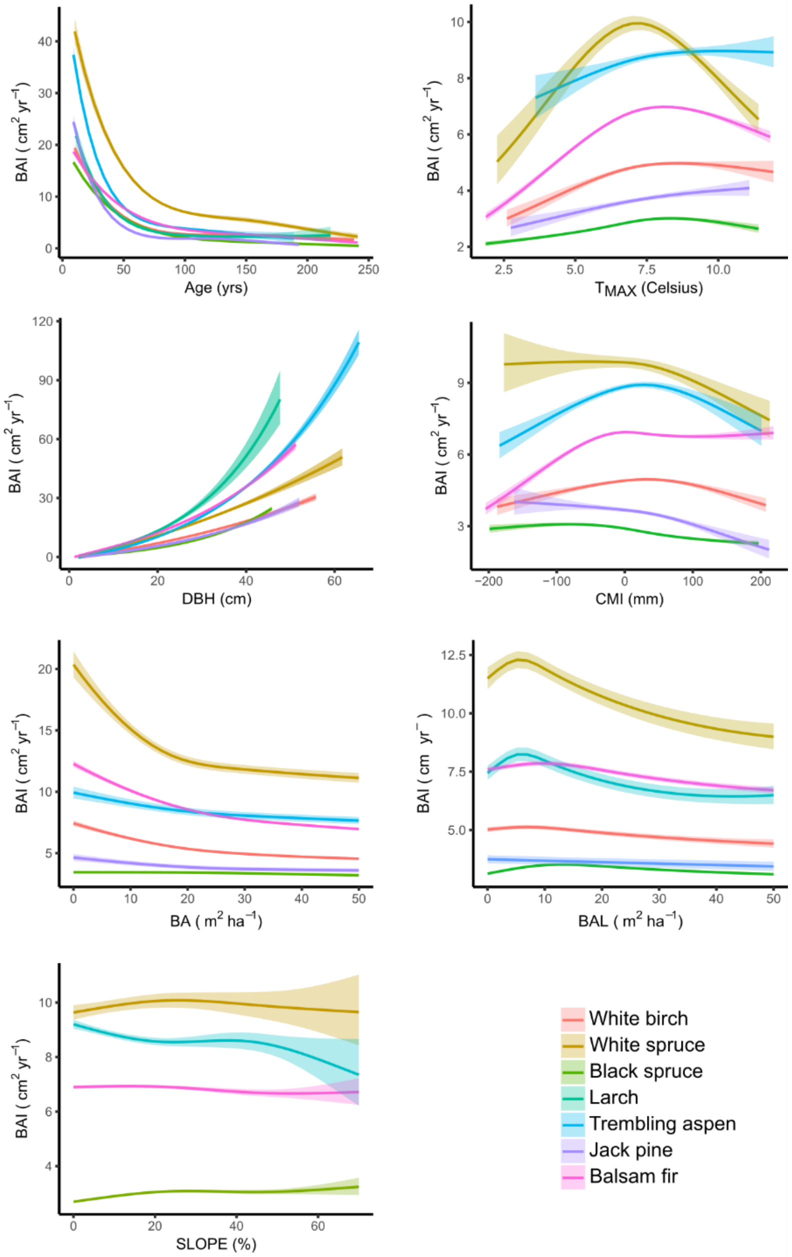

Supplementary Figure 3 Predicted species-specific effect of tree size, slope, tree age, temperature (T_{MAX}), climate moisture index (CMI), symmetric (BA) and asymmetric competition (BAL) on growth (BAI). Shaded areas correspond to the 95% confidence interval. For each plot, all other variables in the model are set to their median value across species. Only significant parameters ($P<0.05$, see Table 1) are displayed.

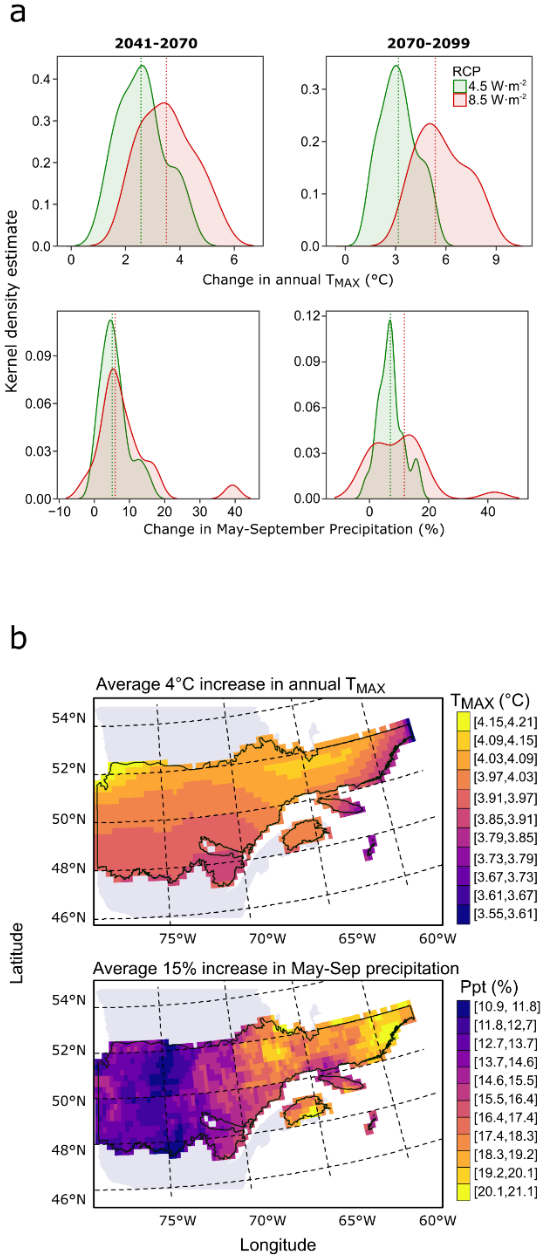

Supplementary Figure 4 **a** Kernel density estimates of future changes in annual T_{MAX} and May to September precipitations averaged over the boreal zone of the study area from 21 climate simulations and two greenhouse gas emission scenarios (RCP) of 4.5 and 8.5 W·m^{-2} (see Methods). Dotted vertical lines indicate median values. **b** Spatial variation corresponding to an average 4°C increase in annual T_{MAX} and 15% increase in May to September precipitation. Data for base maps from https://www12.statcan.gc.ca/census-recensement/2011/geo/bound-limit/bound-limit-2011-eng.cfm with permission under http://open.canada.ca/en/open-government-licence-Canada and from https://www.donneesquebec.ca/recherche/fr/dataset/systeme-hierarchique-de-classification-

ecologique-du-territoire/resource/b336d842-9f1d-4d0e-88c1-d771d8ade785 used with permission under a Creative Commons 4.0 – Attribution CC BY.

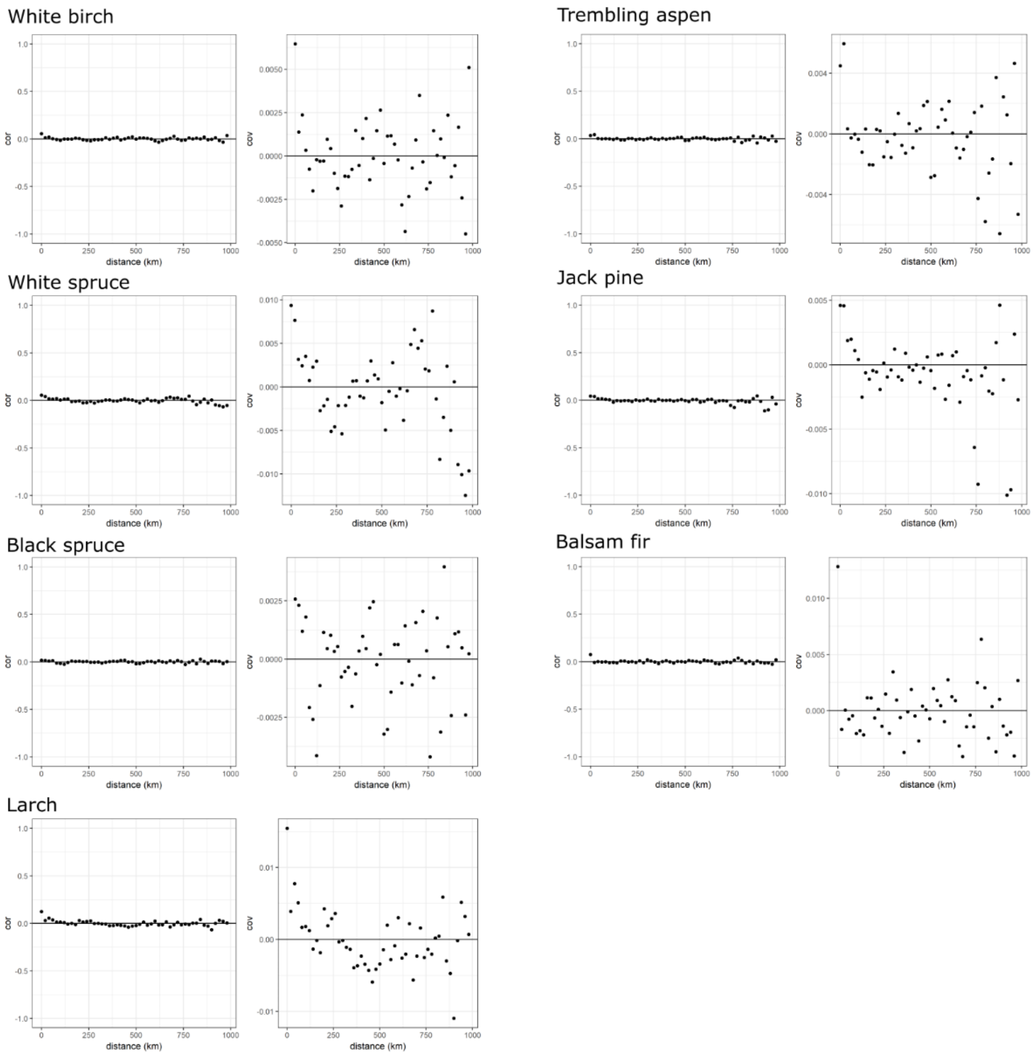

Supplementary Figure 5 Correlation (cor) and covariance (cov) of species-specific model residuals according to distance between sampled plots (binned by 20 km).

Supplementary Table 1. Fraction of the boreal vegetation zone for which warming scenarios fall within the observed range of mean maximum daily temperature (T_{MAX}). Variations among species are due to differences in distribution ranges and analyzed growth periods (10-15 years prior to sampling between 1985-2005).

	**+1°C**	**+2°C**	**+3°C**	**+4°C**
White birch	100%	100%	100%	100%
White spruce	100%	100%	100%	97.3%
Black spruce	100%	100%	100%	98.8%
Aspen	100%	100%	100%	100%
Jack pine	100%	100%	100%	85.2%
Balsam fir	100%	100%	100%	100%

Supplementary Table 2. Average density of stems (DBH>9cm) per hectare by species and stand successional stage (early-seral: 0-20 yrs; immature: 20-70 yrs; mature:70-100 yrs; old-growth: >100 yrs). Different letters indicate significant differences among stand stages at P<0.05. Differences were tested with Tukey HSD post-hoc tests, following significant global one-way anovas (P<0.01).

	**DBH>9cm**			
	**Early-seral**	**Immature**	**Mature**	**Old-growth**
White birch	793 **a**	997 **c**	857 **b**	793 **a**
White spruce	1 102 **a**	960 **b**	740 **c**	708 **c**
Black spruce	925 **a**	1194 **d**	1 117 **c**	1 004 **b**
Larch	893 **a**	1 066 **b**	871 **a**	916 **a**
Aspen	902 **b**	1 064 **a**	835 **c**	735 **d**
Jack pine	1 109 **a**	1 126 **a**	1 129 **a**	815 **b**
Balsam fir	905 **a**	1195 **c**	1 032 **b**	1 074 **b**

Supplementary Table 3. Comparison of general additive models used to analyze basal area increment across species. Explanatory power of different temperature (T_{MEAN}, average daily mean temperature, and T_{MAX}, average daily maximum temperature) and water terms (CMI_{GS}, growing season climate moisture index, and CMI_{SUMMER}, summer climate moisture index) is compared separately and in interaction, as well as explanatory power of all factors in the final multi-species model. Predictions presented in this study are from the global model described here, fitted separately on each species.

		Dev.explained (%)	AIC	Delta AIC
Temperature only	**T_{MAX}**	**28.9**	**393 694**	**-**
	T_{MEAN}	28.6	394 542	848
Water only	**CMI_{GS}**	**23.4**	**411 537**	-
	CMI_{SUMMER}	22.9	412 936	1399
Temperature and water interactions	**T_{MAX} * CMI_{GS}**	**29.3**	**392 198**	**-**
	T_{MAX} * CMI_{SUMMER}	29.3	392 239	41
	T_{MEAN} * CMI_{SUMMER}	29.1	393052	854
	T_{MEAN} * CMI_{GS}	29.0	393093	895
Final model terms	T_{MAX} * CMI_{GS}	**29.3**	**392198**	
	+ log(DBH)	40.1	352352	
	+ DBH	41.8	345366	
	+ Age	61.7	244986	
	+ Slope	61.8	244855	
	+ BA	62.6	239387	
	+ BA * stand stage	62.7	238665	
	+ BAL	63.0	237163	
	+ BAL*stand stage	63.2	235524	
	+ Snow	63.3	235121	
	+ CMI_{GS} *Snow	63.3	234799	
	+ Soil	63.6	232763	
	+ CMI_{GS} *Soil	63.7	232572	
	+ T_{MAX} * Soil	63.7	232482	
	+ Stand stage	63.8	231779	
	+ CMI_{GS}*stand stage	63.8	231671	
	+ T_{MAX} *stand_stage	63.8	231596	

Supplementary Table 4. Influence of soil type (drainage and texture) and stand stage on the slope of the T_{MAX} parameter (temperature sensitivity) for each species growth model. Higher values indicate steeper (positive or negative) slopes.

Factor	Levels	White birch	White spruce	Black spruce	Aspen	Jack pine	Balsam fir
Soil (x10^3)	Shallow	-3.3**	-13.4*	-4.6***	n.s.	-14.0*	n.s.
	Mesic – coarse	6.6**	-23.0*	10.1***	n.s.	-15.1*	n.s.
	Mesic – medium	-7.5**	-10.8*	24.1***	n.s.	11.6*	n.s.
	Mesic – fine	19.1**	-13.6*	-1.8***	n.s.	7.7*	n.s.
	Hygric – coarse	3.6**	13.9*	11.7***	n.s.	-2.2*	n.s.
	Hygric – medium	-6.7**	13.1*	-1.1***	n.s.	18.7*	n.s.
	Hygric - fine	-6.0**	25.3*	-28.9***	n.s.	-25.1*	n.s.
	Hydric	-5.8**	8.5*	-9.5***	n.s.	18.5*	n.s.
Stand Stage (x10^2)	Early-seral	n.s.	5.0 **	-1.2*	n.s.	-1.2*	-1.7***
	Immature	n.s.	0.4**	-0.6*	n.s.	0*	0.2***
	Mature	n.s.	-3.0**	0.9*	n.s.	0.7*	1.0***
	Old-growth	n.s.	-2.3**	1.0*	n.s.	0.6*	0.6***

*: P<0.05; **: P<0.01; ***: P<0.001; n.s.: non-significant

Supplementary Table 5. Influence of soil type (drainage and texture), stand stage and snowfall on the slope of the CMI parameter (water sensitivity) for each species growth model. Higher values indicate steeper (positive or negative) slopes.

**Factor**	**Levels**	**White birch**	**White spruce**	**Black spruce**	**Aspen**	**Jack pine**	**Balsam fir**
Soil (x10^4)	Shallow	n.s.	1.4**	n.s.	n.s.	n.s.	2.5***
	Mesic – coarse	n.s.	-2.6**	n.s.	n.s.	n.s.	0.6***
	Mesic – medium	n.s.	5.5**	n.s.	n.s.	n.s.	3.3***
	Mesic – fine	n.s.	0**	n.s.	n.s.	n.s.	-4.1***
	Hygric – coarse	n.s.	1.5**	n.s.	n.s.	n.s.	-0.8***
	Hygric – medium	n.s.	1.2**	n.s.	n.s.	n.s.	0***
	Hygric - fine	n.s.	-3.6**	n.s.	n.s.	n.s.	-2.2***
	Hydric	n.s.	-3.3**	n.s.	n.s.	n.s.	0.7***
Stand Stage (x10^4)	Early-seral	n.s.	n.s.	-1.3***	2.7**	n.s.	2.2***
	Immature	n.s.	n.s.	-1.6***	2.8**	n.s.	-0.6***
	Mature	n.s.	n.s.	0***	-2.2**	n.s.	0.5***
	Old-growth	n.s.	n.s.	3.0***	-3.4**	n.s.	-2.2***
Snowfall (x10^4)	Low	5.5***	5.0**	-4.0***	0**	-4.5***	-2.1**
	Medium	-3.5***	-2.3**	-1.0***	2.1**	4.1***	1.3**
	high	-2.0***	-2.7**	5.0***	-1.5**	0***	0.8**

*: P<0.05; **: P<0.01; ***: P<0.001; n.s.: non-significant

Supplementary Note 1 - Effects of size and age of growth

The positive tree size effect on growth reported here, regardless of the environment, is well-established across species, continents and forest biomes (tropical, subtropical and temperate)[1,2]. The negative relationship of growth with tree age, while incoherent with the largely documented increasing trend in carbon accumulation with age[1], is consistent with previous studies in similar environments[2,3] and may reflect a sampling bias caused by the absence of old, fast-growing trees and young, slow-growing trees from the tree ring dataset[4-6]. This bias is caused by the fact that fast-growing trees reach their maximum potential size faster and have a shorter lifespan than slow-growing trees[7,8]. As a consequence, sampling only current living trees in a stand for tree ring analyses leads to an underrepresentation of old fast growing trees as they would have died or been harvested before sampling.

Supplementary references

1 Stephenson, N. L. *et al.* Rate of tree carbon accumulation increases continuously with tree size. *Nature* **507**, 90-93 (2014).

2 Foster, J. R., Finley, A. O., D'Amato, A. W., Bradford, J. B. & Banerjee, S. Predicting tree biomass growth in the temperate–boreal ecotone: Is tree size, age, competition, or climate response most important? *Global Change Biology* **22**, 2138-2151 (2016).

3 Buechling, A., Martin, P. H. & Canham, C. D. Climate and competition effects on tree growth in Rocky Mountain forests. *Journal of Ecology* **105**, 1636-1647 (2017).

4 Brienen, R. J. W., Gloor, E. & Zuidema, P. A. Detecting evidence for CO2 fertilization from tree ring studies: The potential role of sampling biases. *Global Biogeochemical Cycles* **26**, GB1025 (2012).

5 Bowman, D. M. J. S., Brienen, R. J. W., Gloor, E., Phillips, O. L. & Prior, L. D. Detecting trends in tree growth: not so simple. *Trends in Plant Science* **18**, 11-17 (2013).

6 Nehrbass-Ahles, C. *et al.* The influence of sampling design on tree-ring-based quantification of forest growth. *Global Change Biology* **20**, 2867-2885 (2014).

7 Di Filippo, A. *et al.* The longevity of broadleaf deciduous trees in Northern Hemisphere temperate forests: insights from tree-ring series. *Frontiers in Ecology and Evolution* **3** (2015).

8 Johnson, S. E. & Abrams, M. D. Age class, longevity and growth rate relationships: protracted growth increases in old trees in the eastern United States. *Tree Physiology* **29**, 1317-1328 (2009).
